# Clinically oriented dual-tier screening for post-stroke epilepsy with interpretable machine learning in a severely imbalanced cohort

**DOI:** 10.3389/fmed.2026.1836846

**Published:** 2026-05-21

**Authors:** Lijun Wu, Han Wu, Lingling Xu, Jingze Li, Jian Wang

**Affiliations:** 1 Fushun People's Hospital, Zigong, China; 2 Department of Clinical Laboratory, Sichuan Clinical Research Center for Cancer, Sichuan Cancer Hospital & Institute, Sichuan Cancer Center, University of Electronic Science and Technology of China, Chengdu, China; 3 College of Information Engineering, Sichuan Agricultural University, Ya'an, China; 4 Department of Neurology, Ya'an People's Hospital, Sichuan Province, Ya' an, China

**Keywords:** epilepsy, machine learning, risk assessment, sensitivity and specificity, stroke

## Abstract

**Background:**

Post-stroke epilepsy is an important complication after stroke, yet its relatively low incidence makes early identification difficult in routine practice. This challenge is compounded by severe class imbalance, which may limit the clinical usefulness of conventional prediction models.

**Methods:**

We conducted a retrospective cohort study of 21,459 patients with stroke, including 936 patients who developed post-stroke epilepsy. Candidate predictors underwent staged feature reduction, after which we developed an interpretable machine-learning framework using an imbalance-aware modeling strategy. Two clinically distinct models were defined within a dual-tier screening framework: a primary model for balanced risk stratification and a secondary model for sensitivity-prioritized alerting. Model interpretation was examined using SHAP.

**Results:**

The cohort showed marked class imbalance (approximately 21.9:1). The primary model achieved the most balanced overall performance, with a macro-area under the curve of 0.996, area under the precision-recall curve of 0.970, F1-score of 0.931, sensitivity of 0.907, and specificity of 0.998. The secondary alert model yielded higher sensitivity (0.971) with lower F1-score (0.854) and specificity (0.985), supporting its role as a high-sensitivity screening tool rather than a general prediction model. Key contributors included neurological severity, hypertension, lactate, D-dimer, and aspartate aminotransferase.

**Conclusion:**

In a severely imbalanced cohort, a clinically oriented dual-tier framework provided both balanced risk stratification and high-sensitivity alerting for post-stroke epilepsy. This approach may support decision-making in follow-up care, although external validation remains necessary.

## 1 Introduction

Post-stroke epilepsy is a clinically meaningful complication that may worsen neurological recovery, increase care burden, and complicate long-term management after stroke (1, 2). Although it is less common than many other post-stroke sequelae, its consequences can be substantial once it occurs (3), particularly in patients who already require close rehabilitation and secondary prevention. This combination of relatively low incidence and high clinical impact makes post-stroke epilepsy an important but difficult target for early recognition. From a practical perspective, timely identification of patients at increased risk may help guide follow-up intensity, patient counseling, and the allocation of limited monitoring resources (4, 5).

Despite growing interest in prediction modeling for post-stroke complications (6–8), post-stroke epilepsy presents a particular methodological difficulty because the outcome is uncommon in comparison with the much larger non-event population (3). In this setting, conventional prediction models may appear satisfactory on global performance metrics while still failing to detect a substantial proportion of patients who later develop epilepsy (9). Such behavior limits clinical value because missed high-risk cases are often the errors of greatest concern. At the same time, an overly aggressive response to imbalance can create a different problem. Artificially forcing near-equal class distributions may improve apparent case detection but can also produce excessive false-positive findings (10), reducing specificity and weakening clinical credibility. A model that labels too many patients as high risk may be statistically interesting yet difficult to justify in real-world follow-up pathways.

Recent work in post-stroke epilepsy prediction has included clinical prognostic scores such as SeLECT, conventional regression-based models, and machine-learning approaches using routinely collected clinical, imaging, laboratory, or multimodal variables (4, 5, 11, 12). Several recent studies have further explored interpretable machine-learning models for post-stroke epilepsy and have identified clinically plausible predictors such as neurological severity, cortical involvement, D-dimer, and other markers of systemic stress (13). In parallel, broader reporting guidance for clinical prediction models has emphasized transparent description of predictor selection, model development, validation, and performance reporting when regression or machine-learning methods are used (14). However, prior approaches have not consistently addressed the combination of severe outcome imbalance, threshold-dependent minority-case detection, false-positive burden, and clinically transparent interpretation. As a result, apparently strong global discrimination can coexist with missed high-risk cases or limited interpretability at the point of use.

These considerations suggest that post-stroke epilepsy prediction should not be framed simply as a search for a single numerically superior classifier. A more clinically useful approach is to acknowledge that different decision contexts may require different operating priorities. In some settings, balanced risk stratification is needed to support general follow-up planning, whereas in others a higher-sensitivity alerting strategy may be preferable when the cost of missed cases is considered greater than the burden of additional false positives. At the same time, interpretability remains essential if model outputs are to be discussed in clinically meaningful terms rather than treated as opaque probabilities (15, 16).

Against this background, we developed a clinically oriented dual-tier screening framework for post-stroke epilepsy in a large retrospective cohort with severe class imbalance. The study was designed to address three practical gaps in prior work: imbalance-aware case detection, clinically interpretable feature reduction, and transparent reporting of threshold-dependent model behavior. Specifically, the framework pairs a balanced risk-stratification model with a sensitivity-prioritized alert model, applies a progressive feature-reduction pipeline, and integrates SHAP-based global, dependence, and individual-level explanations. We also report metric trade-offs and pooled confusion-matrix counts to make the operating behavior of the final models more transparent for clinical interpretation.

## 2 Methods

### 2.1 Study population and data preprocessing

This study was conducted as a retrospective secondary analysis of a publicly available multicenter stroke dataset comprising 21,459 patients, among whom 936 developed post-stroke epilepsy and 20,523 did not, corresponding to a marked class imbalance of approximately 21.9:1 (11, 17). The binary study outcome was the occurrence of post-stroke epilepsy as defined in the curated dataset. Baseline variables were collected from routinely available structured records and covered several clinically relevant domains, including demographic characteristics, vascular comorbidities, lesion location patterns, and laboratory and clinical severity indicators. Representative variables included age, sex, atrial fibrillation, coronary artery disease, parietal or temporal lobe involvement, biomarkers such as glycated hemoglobin, C-reactive protein, albumin, D-dimer, and lactate, and neurological severity as assessed by the National Institutes of Health Stroke Scale. Continuous variables were summarized as medians with interquartile ranges, and categorical variables were summarized as counts with proportions. Because missingness is a recurrent concern in retrospective clinical datasets, a random-forest-based multiple-imputation pipeline was prespecified during preprocessing to ensure methodological completeness (18). However, after the final structured dataset had been loaded and screened at runtime, no detected NaN values were identified in the variables retained for analysis. Accordingly, the imputation procedure was implemented but was not triggered in the present cohort.

### 2.2 Progressive dimensionality reduction

To reduce redundancy and limit overfitting while preserving clinically informative signals, candidate predictors were subjected to a two-stage dimensionality reduction strategy. The initial feature pool contained 74 pre-specified variables. In the first stage, an Elastic Net penalized logistic model was used as a screening tool (19). For coefficient vector β , the penalty was defined as follows. Here, λ denotes the overall regularization strength, α controls the mixture of L1 and L2 shrinkage, and $\beta_{j}$ is the coefficient of the j -th predictor. When α=1 , the penalty becomes LASSO-like, whereas α=0 corresponds to ridge-like regularization. In the present workflow, Elastic Net served as a broad sparse screen that reduced the candidate pool from 74 to 62 variables while stabilizing groups of correlated predictors.

$$P(\beta )=\lambda [\alpha \sum_{j=1}^{p}|\beta_{j}|+\frac{1-\alpha }{2}\sum_{j=1}^{p}\beta_{j}^{2}]$$

In the second stage, the Elastic Net-retained variables were entered into recursive feature elimination with a linear support vector machine under stratified 10-fold cross-validation (20), yielding a final core set of 52 features. Standardization was applied before feature selection and model fitting, and all scaling operations involved in cross-validated procedures were performed independently within each training fold to avoid data leakage into validation data. This progressive strategy was chosen instead of single-step filtering because an isolated filter can discard correlated but clinically meaningful variables too early, whereas principal component analysis reduces dimensionality at the cost of replacing original predictors with latent components. Elastic Net therefore provided a broad interpretable screen, and SVM-RFE supplied recursive refinement within cross-validation. A concise summary of the feature-selection sequence is provided in Supplementary Table S1.

### 2.3 Dual-tier model development

Model development was designed around the central challenge of severe outcome imbalance (9). Because forced 1:1 resampling may generate an unrealistically enlarged minority class and inflate false-positive predictions, a mild adaptive synthetic resampling strategy was employed instead of aggressive balancing (21). Specifically, the minority class was expanded only to 30% of the majority class size after resampling. This design was intended to improve local minority representation while preserving a more realistic class structure. Four comparative models were then evaluated. Model A was a logistic regression baseline, representing a conventional clinical classifier. Model B was a gradient-boosting tree model trained on the original class distribution (22), serving as the principal machine-learning benchmark. Model C combined mild adaptive resampling with the same boosting framework and was prespecified as the primary balanced model because its purpose was general risk stratification rather than maximal recall alone. Model D extended this framework by introducing cost-sensitive learning and a dynamic classification threshold (23), and it was prespecified as the secondary high-sensitivity model for alert-oriented screening.

The cost-sensitive formulation in Model D assigned greater penalty to false-negative errors than to false-positive errors. In simplified form, the weighted binary cross-entropy loss was expressed as

$$ℒ=-\frac{1}{n}\sum_{i=1}^{n}[w_{1}y_{i}\log(p_{i})+w_{0}(1-y_{i})\log(1-p_{i})]$$

where $p_{i}$ denotes the predicted probability for patient i , $w_{0}$ is the non-negative class weight assigned to non-PSE cases, and $w_{1}$ is the non-negative class weight assigned to PSE cases. In the imbalance-aware setting, $w_{1}$ was set greater than $w_{0}$ so that missed positive cases received stronger penalty, but neither weight was treated as an unconstrained fitted parameter. To further align classification with a sensitivity-prioritized clinical objective, the decision threshold for Model D was not fixed at 0.5. Instead, it was optimized within each validation fold according to the Youden criterion (24),

$$\begin{array}{l} J=\max_{c}\{\mathrm{TPR}(c)-\mathrm{FPR}(c)\}\\ =\max_{c}\{\text{Sensitivity}(c)+\text{Specificity}(c)-1\} \end{array}$$

where c is the candidate probability threshold. This procedure identifies the cutoff that maximizes separation between true-positive and false-positive rates on the receiver operating characteristic curve. In the present context, its clinical rationale was to improve interception of high-risk patients in a screening-oriented setting while avoiding an arbitrary universal threshold across distinct decision priorities. Model performance was assessed using stratified 10-fold cross-validation and summarized by accuracy, macro-area under the receiver operating characteristic curve, area under the precision-recall curve, F1-score, sensitivity, and specificity.

### 2.4 Model interpretability and statistical analysis

**Figure 1:**
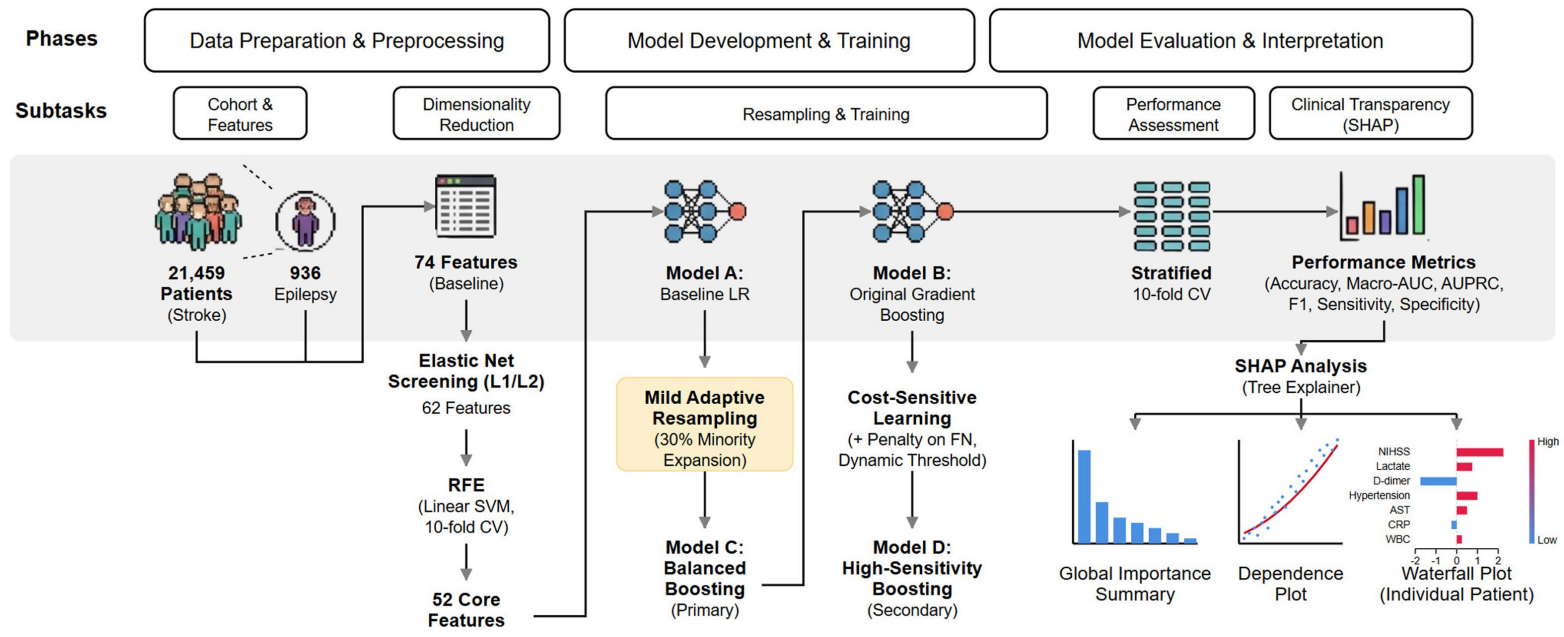
Overall study workflow for the clinically oriented dual-tier prediction framework of post-stroke epilepsy. LR, logistic regression; CV, cross-validation; SHAP, SHapley Additive exPlanations; AUPRC, area under the precision-recall curve; NIHSS, National Institutes of Health Stroke Scale; SVM, support vector machine; RFE, recursive feature elimination.

To improve the clinical transparency of the final framework, *post hoc* interpretation was performed using SHapley Additive exPlanations with the tree-based explainer applied to the primary balanced model (25). For interpretability analysis, the model was refit on the full dataset using the same standardized feature set and the same mild resampling strategy that defined the main balanced model. Explanations were examined at three complementary levels. First, global importance was summarized with SHAP summary visualizations to identify the dominant contributors to predicted post-stroke epilepsy risk across the cohort. Second, dependence plots were generated for leading continuous variables because they show how observed feature values relate to SHAP contributions and help identify nonlinear or threshold-like associations while preserving the original clinical variable scale. Third, waterfall plots were constructed for representative individual patients in order to illustrate how multiple clinical signals jointly shifted predicted risk at the patient level. These visual analyses were intended to convert a statistical prediction rule into a clinically interpretable framework rather than to imply causal inference. Baseline characteristics and model results were reported in manuscript-ready tables, with performance estimates presented as cross-validated summary measures. Taken together, these procedures constituted an integrated, clinically oriented analytical framework that linked cohort preprocessing, progressive dimensionality reduction, dual-tier model construction, and multilevel interpretability analysis within a single workflow. This design was intended to support both methodologic rigor and clinical transparency in post-stroke epilepsy risk assessment, with the overall study workflow summarized in Figure 1.

## 3 Results

### 3.1 Baseline characteristics

The study cohort comprised 21,459 patients, including 936 patients with post-stroke epilepsy and 20,523 without post-stroke epilepsy, corresponding to a marked class imbalance of approximately 21.9:1. Baseline comparisons showed several significant differences between the two groups (Table 1). Patients who developed post-stroke epilepsy had higher neurological severity at presentation, with a median NIHSS score of 11.00 (10.00, 13.00) compared with 7.00 (6.00, 9.00) in those without post-stroke epilepsy. They also showed higher levels of biochemical and inflammatory markers. Median lactate was 2.80 (2.60, 3.00) mmol/L in the post-stroke epilepsy group and 2.40 (2.30, 2.70) mmol/L in the non-epilepsy group, while median D-dimer was 1.99 (1.38, 7.24) ng/mL compared with 0.89 (0.65, 1.42) ng/mL, respectively. C-reactive protein was also higher in patients with post-stroke epilepsy, at 33.50 (20.88, 88.62) mg/L versus 8.90 (4.50, 17.60) mg/L. In addition, cortical lesion distribution differed between groups, with parietal lobe involvement present in 7.26% of patients with post-stroke epilepsy and 2.90% of those without, and temporal lobe involvement present in 6.94 and 2.78%, respectively. Effect-size estimates were added in Supplementary Table S4 to complement *p* values; NIHSS, lactate, D-dimer, C-reactive protein, and parietal or temporal lobe involvement showed the clearest between-group separation. These findings indicated that the post-stroke epilepsy group entered analysis with a profile characterized by greater neurological burden and more pronounced biochemical disturbance.

**Table 1:** Baseline characteristics of the study cohort.

Variable	Overall (*n* = 21,459)	Without PSE (*n* = 20,523)	With PSE (*n* = 936)	*p* value
Age, years	67.00 (59.00, 76.00)	67.00 (59.00, 76.00)	66.00 (56.00, 75.00)	0.006
Male sex, n (%)	10,843 (50.53%)	10,271 (50.05%)	572 (61.11%)	<0.001
Coronary artery disease, n (%)	9,672 (45.07%)	9,328 (45.45%)	344 (36.75%)	<0.001
Atrial fibrillation, n (%)	2043 (9.52%)	1930 (9.40%)	113 (12.07%)	0.009
Parietal lobe involvement, n (%)	663 (3.09%)	595 (2.90%)	68 (7.26%)	<0.001
Temporal lobe involvement, n (%)	636 (2.96%)	571 (2.78%)	65 (6.94%)	<0.001
Subcortical involvement, n (%)	2,443 (11.38%)	2,308 (11.25%)	135 (14.42%)	0.004
HbA1c, %	6.40 (6.00, 7.20)	6.40 (6.00, 7.20)	6.70 (6.10, 7.40)	<0.001
C-reactive protein, mg/L	9.40 (4.70, 19.10)	8.90 (4.50, 17.60)	33.50 (20.88, 88.62)	<0.001
Albumin, g/L	41.20 (39.60, 42.40)	41.20 (39.60, 42.40)	40.90 (38.90, 42.60)	0.022
D-dimer, ng/mL	0.92 (0.65, 1.49)	0.89 (0.65, 1.42)	1.99 (1.38, 7.24)	<0.001
Lactate, mmol/L	2.50 (2.30, 2.70)	2.40 (2.30, 2.70)	2.80 (2.60, 3.00)	<0.001
NIHSS score	8.00 (6.00, 10.00)	7.00 (6.00, 9.00)	11.00 (10.00, 13.00)	<0.001

### 3.2 Progressive feature selection

**Figure 2:**
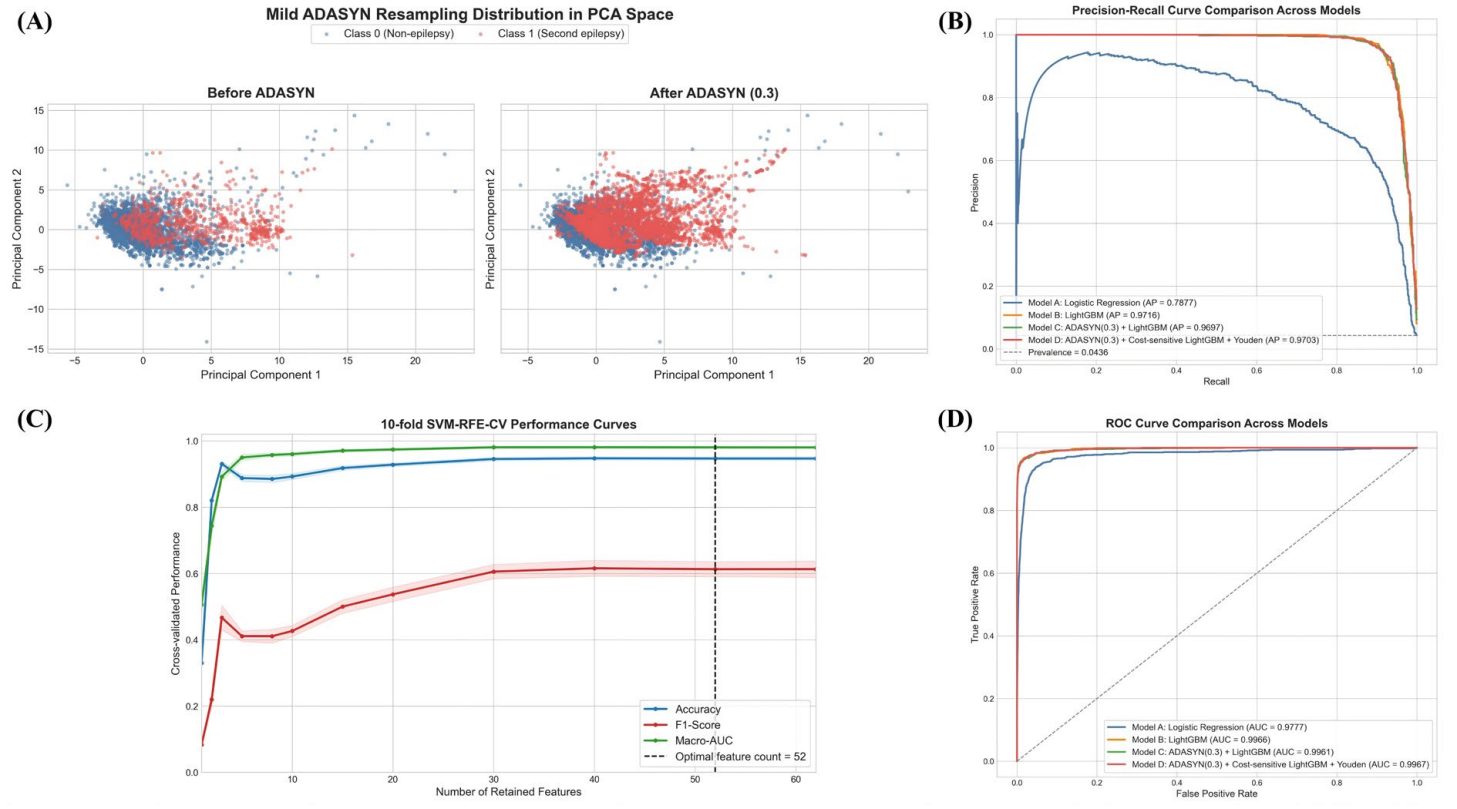
Feature selection, resampling distribution, and comparative model performance. **(A)** Principal component distribution of the majority and minority classes before and after mild adaptive synthetic resampling. **(B)** Precision-recall curves for the four comparative models. **(C)** Ten-fold cross-validated performance curves across retained feature counts during recursive feature elimination. **(D)** Receiver operating characteristic curves for the four comparative models.

The initial predictor pool consisted of 74 pre-specified candidate variables. In the first stage, Elastic Net screening retained 62 variables by shrinking weak coefficients toward zero while preserving correlated signals. These 62 variables were then subjected to recursive feature elimination with support vector machine classification under stratified 10-fold cross-validation, which identified a final set of 52 core features. The stagewise reduction process is summarized in Supplementary Table S1. The cross-validated feature-selection trajectory suggested that model performance improved rapidly during the early phase of feature retention and then entered a relatively stable region as more variables were preserved, with the selected 52-feature solution lying within this plateau rather than at an unstable boundary (Figure 2C). In supplementary comparison against no reduction, principal component analysis, Elastic Net screening alone, and SVM-RFE alone, the progressive Elastic Net plus SVM-RFE strategy achieved comparable discrimination while reducing the predictor space and preserving original clinically interpretable variables (Supplementary Table S2). In parallel, the mild resampling procedure altered minority-class density in principal component space without visually forcing complete class equivalence, which was consistent with the intended preservation of a more realistic underlying class structure (Figure 2A).

### 3.3 Dual-tier model performance

Comparative model evaluation showed clear differences across the four candidate models (Table 2; Figures 2B,D). The logistic regression baseline (Model A) yielded an accuracy of 0.977 ± 0.003, a macro-AUC of 0.978 ± 0.008, an AUPRC of 0.795 ± 0.038, an F1-score of 0.695 ± 0.047, and a sensitivity of 0.596 ± 0.063. These results indicated that, although overall discrimination was acceptable, minority-case detection remained limited under a conventional linear baseline. The conventional boosting model trained on the original class distribution (Model B) improved overall performance, with an accuracy of 0.994 ± 0.001, a macro-AUC of 0.997 ± 0.002, an AUPRC of 0.972 ± 0.009, an F1-score of 0.922 ± 0.015, and a sensitivity of 0.865 ± 0.028. However, its sensitivity remained lower than that of the imbalance-aware models. These metric rankings were not contradictory: under severe imbalance, accuracy mainly reflects the dominant non-event class, macro-AUC reflects threshold-free discrimination, AUPRC is more informative for minority-class retrieval, F1-score captures the precision-recall balance at the selected threshold, and sensitivity or specificity emphasize different clinical error costs.

**Table 2:** Performance comparison of candidate models across 10-fold cross-validation.

Model	Accuracy	Macro-AUC	AUPRC	F1-score	Sensitivity	Specificity
A. LR	0.977 ± 0.003	0.978 ± 0.008	0.795 ± 0.038	0.695 ± 0.047	0.596 ± 0.063	0.995
B. LGBM	0.994 ± 0.001	0.997 ± 0.002	0.972 ± 0.009	0.922 ± 0.015	0.865 ± 0.028	0.999
C. ADASYN + LGBM	0.994 ± 0.001	0.996 ± 0.002	0.970 ± 0.009	0.931 ± 0.017	0.907 ± 0.030	0.998
D. ADASYN + CS-LGBM + Youden	0.985 ± 0.010	0.997 ± 0.001	0.970 ± 0.009	0.854 ± 0.082	0.971 ± 0.010	0.985

Within the dual-tier framework, Model C was designated as the primary balanced model. It achieved an accuracy of 0.994 ± 0.001, a macro-AUC of 0.996 ± 0.002, an AUPRC of 0.970 ± 0.009, an F1-score of 0.931 ± 0.017, a sensitivity of 0.907 ± 0.030, and a specificity of 0.998. Although Model B showed numerically similar discrimination, Model C provided a more favorable balance between case detection and classification stability, particularly through the combination of higher sensitivity and the highest F1-score among the four models. This performance profile supported its use for routine risk stratification, where both missed cases and excessive false-positive labeling are clinically relevant concerns. A supplementary Random Forest comparator showed strong discrimination but lower sensitivity and F1-score than the primary balanced model, and therefore did not alter the main A-D model interpretation (Supplementary Table S3).

Model D was designed as the secondary alert model for settings in which sensitivity was prioritized. It achieved an accuracy of 0.985 ± 0.010, a macro-AUC of 0.997 ± 0.001, an AUPRC of 0.970 ± 0.009, an F1-score of 0.854 ± 0.082, a sensitivity of 0.971 ± 0.010, and a specificity of 0.985. This model yielded the highest sensitivity among all candidates, but this gain occurred alongside lower specificity and a lower F1-score than Model C. The precision-recall and receiver operating characteristic curves were consistent with this interpretation, showing preserved global discrimination while reflecting the distinct operating priorities of the balanced and sensitivity-oriented models (Figures 2B,D). Pooled out-of-fold confusion-matrix counts further clarified the trade-off, with Model C favoring balanced risk stratification and Model D favoring missed-case reduction at the cost of more false positives (Table 3). Taken together, these results supported a clinically differentiated strategy in which Model C served as the principal model for balanced risk assessment and Model D served as a secondary model for high-sensitivity alerting.

**Table 3:** Confusion-matrix summary of the dual-tier models.

Model	TP	FP	TN	FN	Sensitivity	Specificity	Precision	F1-score
Primary balanced model	849	39	20,484	87	0.907	0.998	0.956	0.931
Secondary alert model	909	302	20,221	27	0.971	0.985	0.751	0.847

Primary balanced model: ADASYN(0.3) + LightGBM. Secondary alert model: ADASYN(0.3) + cost-sensitive LightGBM with Youden-threshold tuning.

### 3.4 Model interpretability and clinical signals

**Figure 3:**
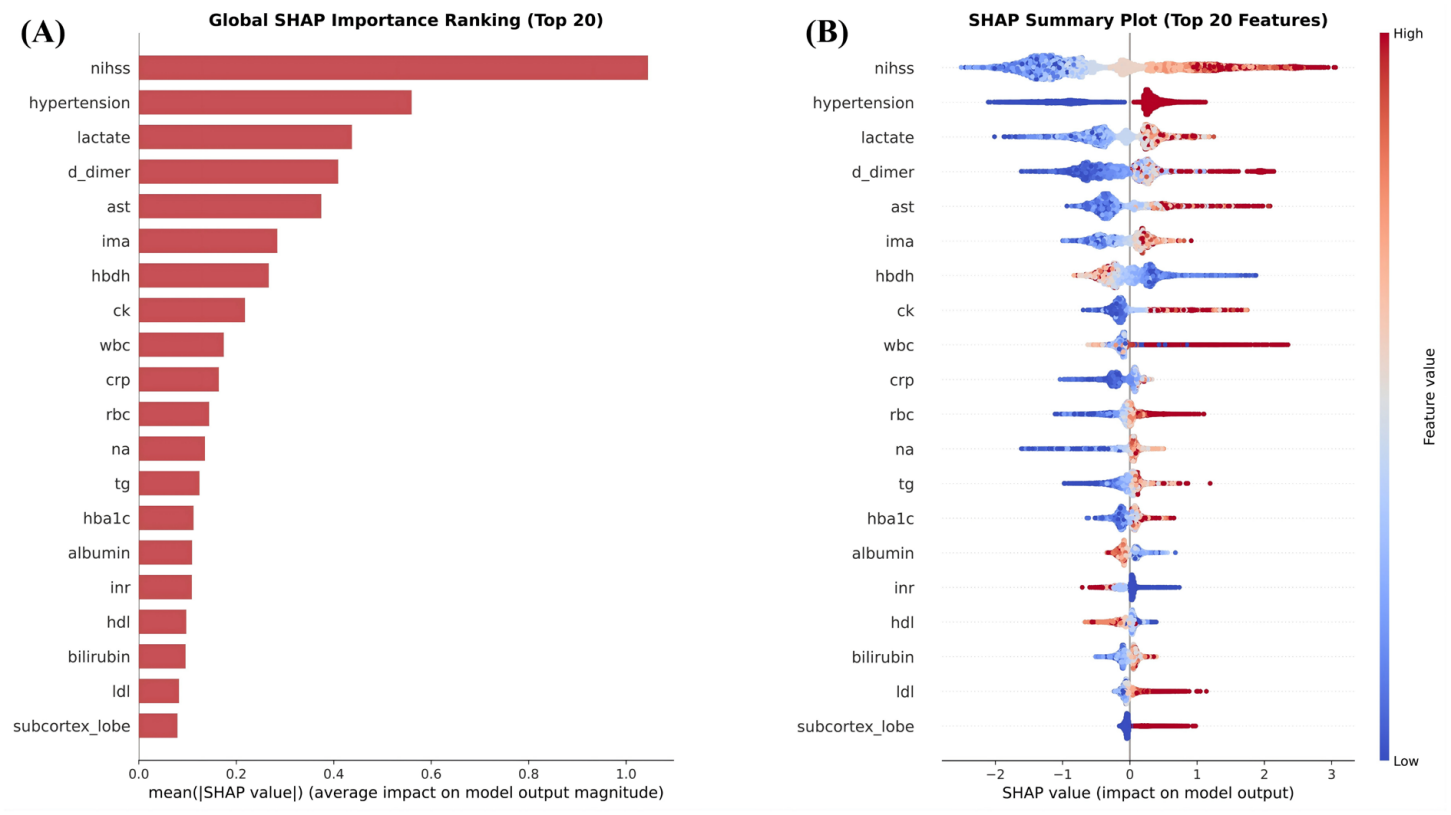
Global SHAP summary of the primary balanced model. **(A)** Mean absolute SHA*p* values. **(B)** SHAP summary distribution.

SHAP analysis showed that the primary balanced model was driven by a clinically coherent group of predictors. In the global importance ranking, NIHSS had the largest overall contribution, followed by hypertension, lactate, D-dimer, and aspartate aminotransferase (Figure 3A). The summary distribution further showed that higher values of NIHSS, lactate, D-dimer, and aspartate aminotransferase were generally associated with positive SHAP values and therefore with higher model-assigned probability of post-stroke epilepsy, whereas lower values tended to shift the prediction in the opposite direction (Figure 3B). Hypertension also showed a consistent positive contribution pattern in patients assigned higher predicted risk. Taken together, these global findings indicated that the model gave substantial weight to neurological severity, vascular comorbidity burden, and biochemical stress-related signals.

**Figure 4:**
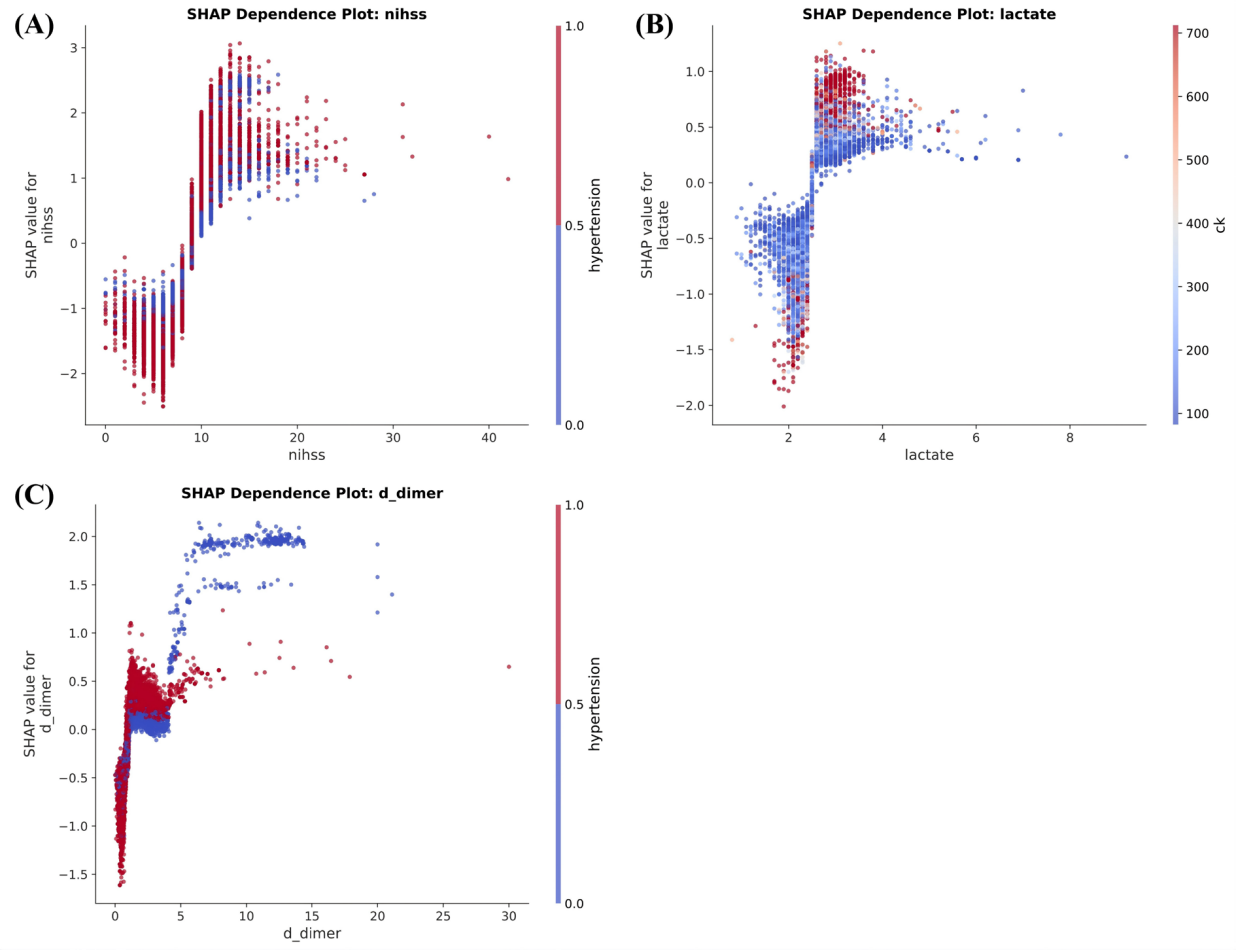
SHAP dependence plots for key continuous predictors. **(A)** NIHSS score. **(B)** Lactate. **(C)** D-dimer.

The dependence plots showed that these associations were not strictly linear. In Figure 4A, increasing NIHSS was associated with progressively larger positive SHAP contributions over the higher range of observed scores, suggesting that greater neurological deficit shifted the model toward higher predicted post-stroke epilepsy risk. In Figure 4B, lactate showed a transition from negative or near-neutral SHAP contributions toward positive values as measurements increased, consistent with a model response to increasing metabolic stress. A similar upward pattern was observed for D-dimer in Figure 4C, where higher values corresponded to more positive SHAP contributions, consistent with coagulation activation and broader stroke-severity burden captured by the model. These plots suggested that the model incorporated threshold-like and nonlinear effects rather than relying on simple linear relationships. These patterns should be interpreted as model-based associations rather than causal effects.

**Figure 5:**
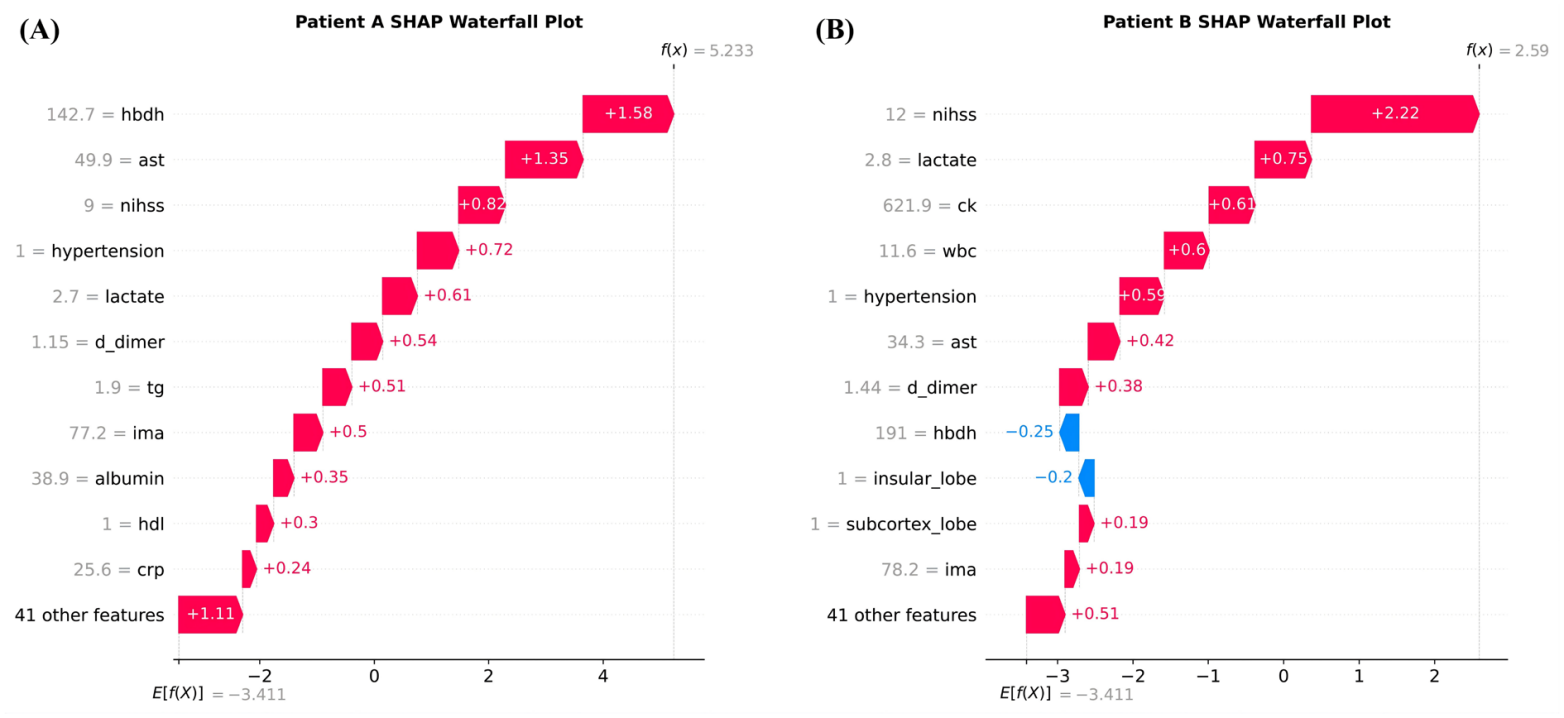
Representative individual SHAP waterfall plots. **(A)** Patient A. **(B)** Patient B.

Patient-level attribution analysis provided further detail regarding how these signals were combined in individual predictions. In the representative true-positive case shown in Figure 5A, the prediction was increased jointly by hydroxybutyrate dehydrogenase, aspartate aminotransferase, NIHSS, hypertension, lactate, D-dimer, triglycerides, ischemia-modified albumin, albumin, high-density lipoprotein, and C-reactive protein, indicating that the final risk estimate arose from the accumulation of multiple concordant abnormalities rather than from a single dominant feature. In the representative high-scoring false-positive case shown in Figure 5B, the model output was driven upward primarily by NIHSS, lactate, creatine kinase, white blood cell count, hypertension, aspartate aminotransferase, and D-dimer, while hydroxybutyrate dehydrogenase and insular lobe involvement contributed in the opposite direction. This pattern indicated that even when the observed outcome was negative, the model still responded to a multidimensional profile characterized by neurological severity, coagulation-related markers, and biochemical stress signals.

## 4 Discussion

In this retrospective secondary analysis of a publicly available multicenter cohort of 21,459 patients, we developed and interpreted a machine-learning framework for risk assessment under conditions of severe outcome imbalance. The central finding was that acceptable prediction in this setting did not depend solely on maximizing a single summary metric (5), but rather on balancing discrimination, case detection, and clinical usability. Within this framework, the primary balanced model achieved a macro-AUC of 0.996 ± 0.002, an AUPRC of 0.970 ± 0.009, an F1-score of 0.931 ± 0.017, and a sensitivity of 0.907 ± 0.030, whereas the secondary alert model increased sensitivity to 0.971 ± 0.010 at the cost of lower specificity and a lower F1-score. This pattern supported the view that post-stroke epilepsy prediction in an imbalanced cohort is better approached as a dual-tier clinical task than as a competition to identify a single universally optimal classifier (26). The additional incorporation of SHAP-based interpretation further allowed the model outputs to be examined in relation to recognizable clinical and biological domains rather than as isolated algorithmic probabilities.

This dual-tier structure has practical implications because post-stroke epilepsy is both relatively infrequent and clinically consequential. In such a context, a single operating threshold may be difficult to justify across all care settings (27, 28). A model tuned too conservatively may miss patients who warrant closer surveillance, whereas a model tuned too aggressively may label too many patients as high risk and thereby weaken its usefulness in routine care. The present results suggest that these competing objectives may be better separated than forced into one decision rule. The secondary alert model, which yielded the highest sensitivity, may be useful in settings where initial screening is intended to minimize missed cases (16), such as early inpatient evaluation or first-line post-stroke assessment, where the clinical consequence of overlooking an evolving high-risk patient may outweigh the burden of additional false-positive alerts. By contrast, the primary balanced model, which achieved the highest F1-score and maintained very high specificity, may be better suited to downstream risk stratification during ward management or outpatient follow-up, where a more selective approach may help guide the allocation of limited monitoring resources such as electroencephalography, repeat neurological assessment, or intensified follow-up scheduling. In this sense, the study does not suggest that one model should replace the other. Rather, it suggests that different models may support different stages of clinical decision-making within the same disease process.

The interpretability analysis also supported the biological plausibility of the framework. NIHSS emerged as the strongest global contributor (4, 12, 29), which is consistent with the view that greater neurological deficit may reflect more extensive brain injury, a larger burden of cortical dysfunction, and a more permissive substrate for abnormal neuronal excitability. Lactate and D-dimer were also among the most influential features. Elevated lactate may reflect metabolic stress, impaired perfusion (30), tissue hypoxia, and systemic stress responses in the acute stroke setting, all of which could plausibly accompany more severe cerebral injury and a greater likelihood of epileptogenic vulnerability. Elevated D-dimer may indicate activation of coagulation pathways, microcirculatory disturbance, and secondary tissue injury, mechanisms that may coexist with blood–brain barrier disruption and inflammatory responses relevant to epileptogenesis (31, 32). Hypertension and aspartate aminotransferase also ranked prominently, further suggesting that vascular burden and biochemical stress were integrated into the prediction process (30). Importantly, the dependence plots showed that these relationships were not purely linear. Higher NIHSS, lactate, and D-dimer values were associated with progressively more positive contributions to model output, indicating that the algorithm captured graded and threshold-like risk patterns rather than responding to random variation. These findings do not establish causality, but they do suggest that the model learned clinically coherent signals rather than relying primarily on statistical noise (33).

An additional point of interest was the behavior of the model in representative false-positive cases. In the high-scoring false-positive patient shown in the individual attribution analysis, the prediction was driven upward by a cluster of abnormalities that included higher NIHSS, lactate, creatine kinase, white blood cell count, hypertension, aspartate aminotransferase, and D-dimer. From a conventional classification perspective, such cases contribute to reduced precision. However, a strictly binary interpretation may be incomplete in a disease process such as post-stroke epilepsy, in which biological vulnerability may precede clinically recognized events. It is therefore possible that some high-scoring false-positive cases represented patients with a subclinical epileptogenic state or a profile of heightened neurological and systemic stress that was not captured by the observed endpoint definition (5, 30). This interpretation should be approached cautiously, because the present data do not permit confirmation of occult epilepsy or delayed seizure emergence. Nevertheless, the clustering of pathophysiologically plausible signals in these cases suggests that at least some false-positive outputs may reflect clinically relevant risk enrichment rather than arbitrary error alone. If this possibility is confirmed in future studies with more detailed longitudinal phenotyping, such cases could become particularly informative for targeted surveillance or early preventive strategies.

Several limitations should be considered when interpreting these findings. First, the study was based on a retrospective secondary analysis of a publicly available multicenter dataset, and limitations related to the original data source, patient selection, and center-specific practice patterns may have influenced the recorded predictors and observed outcome distribution. Second, no external validation cohort was available, and the generalizability of the present framework therefore remains uncertain. The model should be regarded as hypothesis-generating and decision-supportive rather than ready for unrestricted clinical deployment (15). Third, the analysis addressed risk classification rather than the timing of post-stroke epilepsy onset. Because no time-to-event modeling was performed, the study could not estimate when epilepsy might occur after stroke, which limits temporal clinical interpretation. Fourth, the threshold optimization strategy used for the sensitivity-oriented model was data-dependent, and the Youden-based cutoff identified within cross-validation may not transfer directly to other populations with different event rates or care pathways. Additional limitations include the absence of formal calibration analysis in the present report and the inherent constraints of structured retrospective data, which may not fully capture electroclinical nuance. Future work should therefore focus on multicenter external validation, prospective assessment in real clinical workflows, calibration analysis (5), and longitudinal modeling that incorporates time-dependent information. Such studies will be necessary to determine whether the dual-tier strategy retains its performance and clinical relevance across broader populations and whether its outputs can be integrated into follow-up protocols in a way that improves care efficiency without increasing unnecessary intervention.

## 5 Conclusion

In this retrospective secondary analysis of a publicly available multicenter dataset, we developed an interpretable machine-learning framework for post-stroke epilepsy risk assessment in a severely imbalanced cohort and showed that a dual-tier strategy may be more clinically useful than a single-model approach. The primary model provided balanced risk stratification, whereas the secondary model prioritized sensitivity for early alerting when missed cases were of greater concern. The dominant predictors identified by SHAP, including neurological severity, metabolic stress, and coagulation-related markers, were clinically plausible and supported the biological coherence of the framework. From a practical perspective, this dual-tier design could potentially assist clinicians in allocating monitoring resources more rationally, such as prioritizing prolonged video electroencephalographic monitoring for higher-risk patients and adjusting the intensity or frequency of outpatient follow-up after stroke. External validation and prospective multicenter evaluation remain necessary before broader clinical implementation.

## Data Availability

Publicly available datasets were analyzed in this study. This data can be found at: https://doi.org/10.5061/dryad.w0vt4b92c.
