# Supplementary material for: Clinically oriented dual-tier screening for post-stroke epilepsy with interpretable machine learning in a severely imbalanced cohort
Source: Front Med (Lausanne). 2026 May 21;13:1836846. doi: 10.3389/fmed.2026.1836846 (PMC13233222; doi:10.3389/fmed.2026.1836846)
Supplement: Supplementary file 1 [file Table_1.DOCX]

**Supplementary Table S1.** Feature selection summary.

| **Stage** | **Method** | **Features retained** |
| --- | --- | --- |
| **Initial candidate set** | All pre-specified predictors | 74 |
| **Stage 1** | Elastic Net screening | 62 |
| **Stage 2** | SVM-RFE-CV | 52 |
