# Supplementary material for: Clinically oriented dual-tier screening for post-stroke epilepsy with interpretable machine learning in a severely imbalanced cohort
Source: Front Med (Lausanne). 2026 May 21;13:1836846. doi: 10.3389/fmed.2026.1836846 (PMC13233222; doi:10.3389/fmed.2026.1836846)
Supplement: Supplementary file 2 [file Table_2.DOCX]

**Supplementary Table S2.** Comparison of feature-reduction strategies.

| **Strategy** | **Feature form** | **No.** | **Accuracy** | **Macro-AUC** | **AUPRC** | **F1-score** | **Sensitivity** | **Specificity** |
| --- | --- | --- | --- | --- | --- | --- | --- | --- |
| **No reduction** | Original variables | 74 | 0.995 ± 0.001 | 0.997 ± 0.002 | 0.974 ± 0.009 | 0.936 ± 0.018 | 0.906 ± 0.031 | 0.999 ± 0.001 |
| **PCA** | Components | 53 | 0.988 ± 0.002 | 0.991 ± 0.004 | 0.925 ± 0.021 | 0.862 ± 0.029 | 0.842 ± 0.048 | 0.995 ± 0.001 |
| **Elastic Net** | Original variables | 62 | 0.994 ± 0.001 | 0.996 ± 0.002 | 0.970 ± 0.009 | 0.930 ± 0.014 | 0.906 ± 0.026 | 0.998 ± 0.001 |
| **SVM-RFE** | Original variables | 52 | 0.995 ± 0.001 | 0.997 ± 0.002 | 0.972 ± 0.008 | 0.936 ± 0.017 | 0.911 ± 0.036 | 0.998 ± 0.001 |
| **Elastic Net + SVM-RFE** | Original variables | 52 | 0.994 ± 0.001 | 0.996 ± 0.002 | 0.970 ± 0.009 | 0.931 ± 0.017 | 0.907 ± 0.030 | 0.998 ± 0.001 |

Note: Values are mean ± standard deviation from 10-fold cross-validation. “No.” indicates the number of retained predictors or components. PCA, principal component analysis; SVM-RFE, support vector machine recursive feature elimination. The Elastic Net + SVM-RFE strategy was retained because it provided comparable performance while reducing the predictor space and preserving original clinically interpretable variables.
