# Supplementary material for: Clinically oriented dual-tier screening for post-stroke epilepsy with interpretable machine learning in a severely imbalanced cohort
Source: Front Med (Lausanne). 2026 May 21;13:1836846. doi: 10.3389/fmed.2026.1836846 (PMC13233222; doi:10.3389/fmed.2026.1836846)
Supplement: Supplementary file 3 [file Table_3.DOCX]

**Supplementary Table S3.** Random Forest supplementary baseline.

| **Model** | **Retained predictors** | **Accuracy** | **Macro-AUC** | **AUPRC** | **F1-score** | **Sensitivity** | **Specificity** |
| --- | --- | --- | --- | --- | --- | --- | --- |
| **Logistic regression** | 52 | 0.977 ± 0.003 | 0.978 ± 0.008 | 0.795 ± 0.038 | 0.695 ± 0.047 | 0.596 ± 0.063 | 0.995 |
| **Random Forest** | 52 | 0.991 ± 0.001 | 0.998 ± 0.001 | 0.978 ± 0.009 | 0.885 ± 0.015 | 0.797 ± 0.023 | 1.000 ± 0.000 |
