# Supplementary material for: Clinically oriented dual-tier screening for post-stroke epilepsy with interpretable machine learning in a severely imbalanced cohort
Source: Front Med (Lausanne). 2026 May 21;13:1836846. doi: 10.3389/fmed.2026.1836846 (PMC13233222; doi:10.3389/fmed.2026.1836846)
Supplement: Supplementary file 4 [file Table_4.DOCX]

**Supplementary Table S4.** Effect-size estimates for baseline variables.

| **Variable** | **Effect measure** | **Estimate** | **95% CI** |
| --- | --- | --- | --- |
| Age | Cohen's d | -0.128 | Not applicable |
| Platelet count | Cohen's d | -0.348 | Not applicable |
| White blood cell count | Cohen's d | 2.488 | Not applicable |
| Red blood cell count | Cohen's d | 0.335 | Not applicable |
| HbA1c | Cohen's d | 0.118 | Not applicable |
| C-reactive protein | Cohen's d | 1.91 | Not applicable |
| Triglycerides | Cohen's d | 0.297 | Not applicable |
| Low-density lipoprotein | Cohen's d | 0.007 | Not applicable |
| High-density lipoprotein | Cohen's d | -0.031 | Not applicable |
| Aspartate aminotransferase | Cohen's d | 0.94 | Not applicable |
| Alanine aminotransferase | Cohen's d | 0.265 | Not applicable |
| Bilirubin | Cohen's d | 0.333 | Not applicable |
| Albumin | Cohen's d | -0.067 | Not applicable |
| Urea | Cohen's d | -0.062 | Not applicable |
| Creatinine | Cohen's d | -0.021 | Not applicable |
| Blood uric acid | Cohen's d | -0.02 | Not applicable |
| Prothrombin time | Cohen's d | -0.023 | Not applicable |
| Activated partial thromboplastin time | Cohen's d | -0.28 | Not applicable |
| Thrombin time | Cohen's d | 0.288 | Not applicable |
| International normalized ratio | Cohen's d | -0.061 | Not applicable |
| D-dimer | Cohen's d | 2.403 | Not applicable |
| Fibrinogen | Cohen's d | -0.161 | Not applicable |
| Creatine kinase | Cohen's d | 2.585 | Not applicable |
| Creatine kinase-MB | Cohen's d | 1.397 | Not applicable |
| Lactate dehydrogenase | Cohen's d | 1.049 | Not applicable |
| Hydroxybutyrate dehydrogenase | Cohen's d | 0.689 | Not applicable |
| Ischemia-modified albumin | Cohen's d | 0.483 | Not applicable |
| Na | Cohen's d | -0.104 | Not applicable |
| K | Cohen's d | -0.063 | Not applicable |
| Cl | Cohen's d | -0.411 | Not applicable |
| Ca | Cohen's d | 0.143 | Not applicable |
| P | Cohen's d | 0.175 | Not applicable |
| Lactate | Cohen's d | 0.779 | Not applicable |
| Anion gap | Cohen's d | 0.511 | Not applicable |
| Total carbon dioxide | Cohen's d | -0.042 | Not applicable |
| NIHSS score | Cohen's d | 1.283 | Not applicable |
| Uremia | Odds ratio | 2.466 | 1.528 to 3.980 |
| Deep vein thrombosis | Odds ratio | 1.932 | 1.560 to 2.392 |
| Fatty liver disease | Odds ratio | 0.71 | 0.591 to 0.851 |
| Diabetes mellitus | Odds ratio | 1.049 | 0.914 to 1.203 |
| Hypertension | Odds ratio | 1.057 | 0.917 to 1.219 |
| Coronary artery disease | Odds ratio | 0.697 | 0.609 to 0.799 |
| Atrial fibrillation | Odds ratio | 1.323 | 1.081 to 1.619 |
| Cerebral herniation | Odds ratio | 2.761 | 1.727 to 4.416 |
| Hydrocephalus | Odds ratio | 6.013 | 4.470 to 8.091 |
| Hyperuricemia | Odds ratio | 1.605 | 1.341 to 1.921 |
| Hyperlipidaemia | Odds ratio | 0.716 | 0.599 to 0.855 |
| Hypoproteinemia | Odds ratio | 1.885 | 1.591 to 2.234 |
| Frontal lobe involvement | Odds ratio | 2.291 | 1.810 to 2.901 |
| Parietal lobe involvement | Odds ratio | 2.624 | 2.023 to 3.403 |
| Temporal lobe involvement | Odds ratio | 2.608 | 2.000 to 3.400 |
| Occipital lobe involvement | Odds ratio | 2.105 | 1.472 to 3.011 |
| Insular lobe involvement | Odds ratio | 1.25 | 0.740 to 2.112 |
| Multilobar cortical involvement | Cramer's V | 0.063 | Not applicable |
| Basal ganglia involvement | Odds ratio | 1.424 | 1.083 to 1.872 |
| Internal capsule involvement | Odds ratio | 0.755 | 0.045 to 12.670 |
| Brainstem involvement | Odds ratio | 1.377 | 0.827 to 2.291 |
| Cerebellar involvement | Odds ratio | 0.929 | 0.577 to 1.497 |
| Periventricular involvement | Odds ratio | 1.204 | 0.908 to 1.597 |
| Centrum semiovale involvement | Odds ratio | 1.221 | 0.850 to 1.755 |
| Thalamic involvement | Odds ratio | 1.707 | 1.038 to 2.808 |
| Anterior cerebral artery involvement | Odds ratio | 1.093 | 0.624 to 1.916 |
| Middle cerebral artery involvement | Odds ratio | 1.149 | 0.833 to 1.584 |
| Posterior cerebral artery involvement | Odds ratio | 0.73 | 0.178 to 2.992 |
| Vertebral artery involvement | Odds ratio | 0.946 | 0.640 to 1.398 |
| Basilar artery involvement | Odds ratio | 1.085 | 0.554 to 2.126 |
| Male sex | Odds ratio | 1.569 | 1.371 to 1.794 |
| Common carotid artery plaque | Odds ratio | 0.935 | 0.797 to 1.096 |
| Internal carotid artery plaque | Odds ratio | 1.22 | 0.959 to 1.552 |
| External carotid artery plaque | Odds ratio | 1.273 | 0.691 to 2.345 |
| Subcortical involvement | Odds ratio | 1.33 | 1.103 to 1.604 |
| Anterior circulation involvement | Odds ratio | 1.117 | 0.867 to 1.439 |
| Posterior circulation involvement | Odds ratio | 0.855 | 0.722 to 1.011 |
| Large-vessel atherosclerosis | Odds ratio | 0.965 | 0.829 to 1.122 |
